# Supplementary material for: Vaccinating Children against SARS-CoV-2: A Literature Review and Survey of International Experts to Assess Safety, Efficacy and Perceptions of Vaccine Use in Children
Source: Vaccines (Basel). 2022 Dec 29;11(1):78. doi: 10.3390/vaccines11010078 (PMC9862079; doi:10.3390/vaccines11010078)
Supplement: Supplementary file 1 [file vaccines-11-00078-s001.zip › vaccines-2001635-supplementary.pdf]

Table of Contents

Search terms and strategy ***1***

Prisma Flow charts ***2***

Data items ***6***

Forrest plots ***6***

Search terms and strategy

Databases searched

The following databases were searched; EMBASE, PubMed, Cochrane and Clinicaltrials.gov. Previous systematic reviews were reviewed, and all included trials were cross-checked against our own search.

Search terms

The search terms can be seen in table S1.

Table S1 Search terms used

Area	Keywords
Pediatric trial data – efficacy data	SARS / COVID /CORONA<br>PAEDIATRIC / PEDIATRIC / CHILD* / ADOLESCE* / INFANT<br>VACCIN*<br>TRIAL<br>EFFICACY / EFFECTIVE*
Pediatric safety data including clinical trials and post-marketing surveillance	SARS / COVID / CORONA<br>PAEDIATRIC / PEDIATRIC / CHILD* / ADOLESCE* / INFANT<br>TRIAL<br>VACCIN*<br>SAFE*

Prisma Flow charts

Figure S1 Prisma flow chart - safety

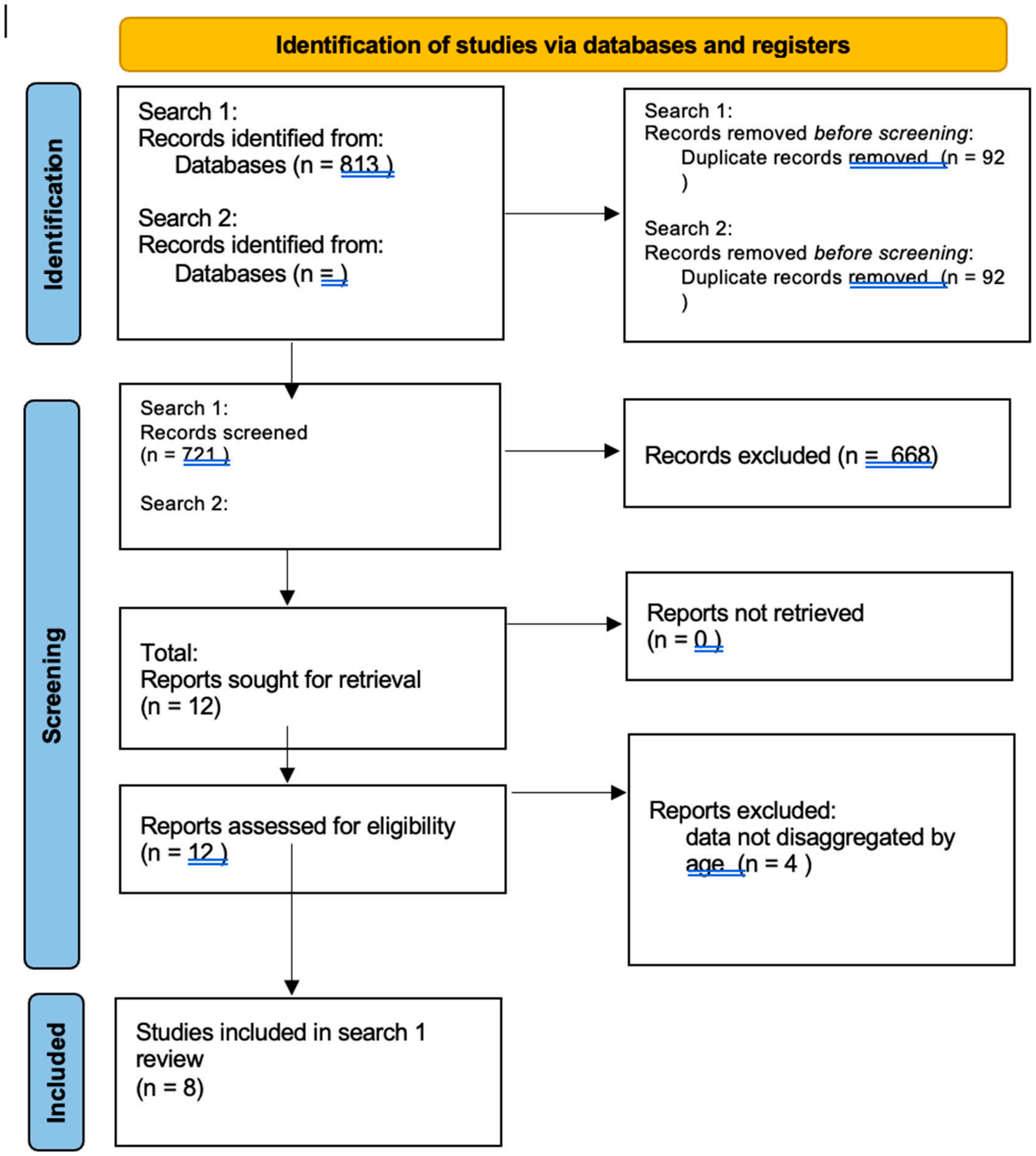

Figure S2: Immunogencity and Efficacy 1

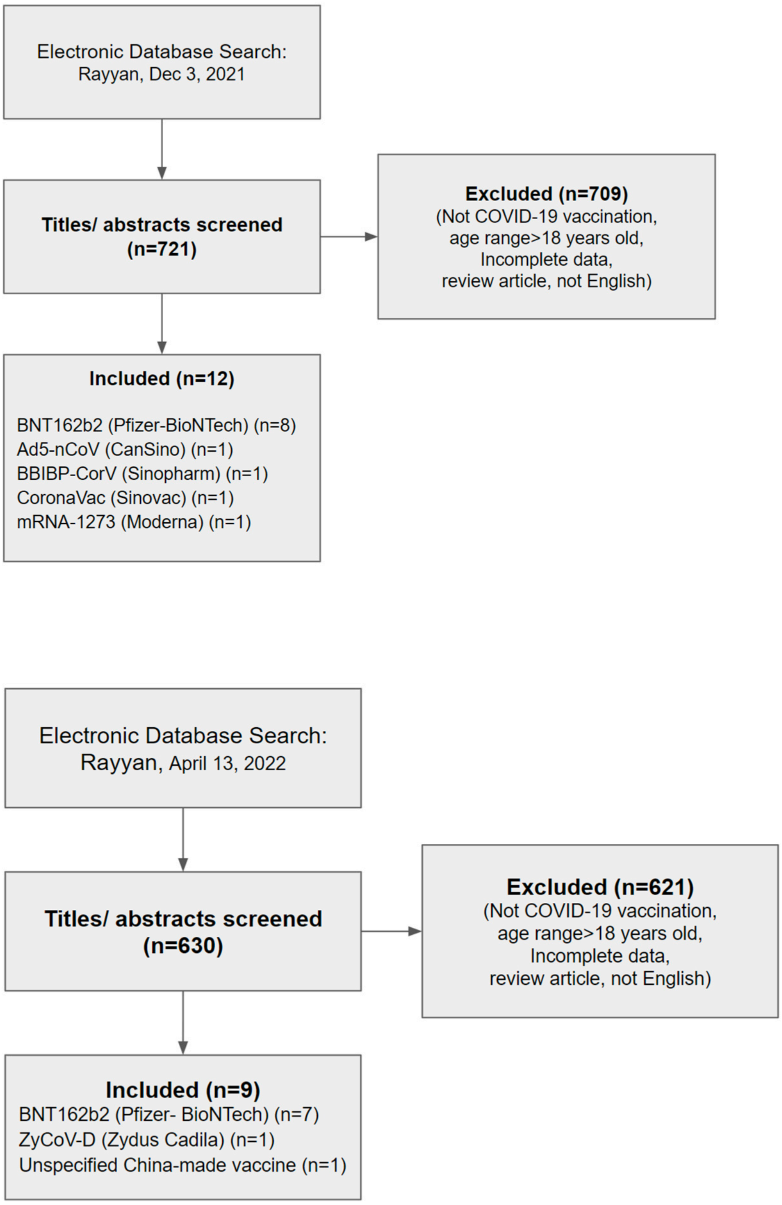

Data items

The following data were extracted where available for all studies which met inclusion criteria; first author, age of study subjects, study type, intervention measures (vaccine type, number of doses, etc), and trial location.

For studies included in the safety review the following information was also obtained: period of trial (as a rough analogue to predominant circulating SARS-CoV-2 strain), adverse events (including definitions, solicited, unsolicited, and serious). Given the heterogeneity of reported adverse effects, all of those listed in the primary paper and any supplemental material were extracted to allow comparison between studies. Data which was not presented with a numerical count or clear percentage (i.e., a bar chart with no data figures elsewhere in the text) was excluded.

Forrest plots

Figure S3 Erythema dose 1 and 2

Study or Subgroup	Vaccine Events	Vaccine Total	Placebo Events	Placebo Total	Weight	Odds Ratio M-H, Fixed, 95% CI
Ali et al	336	2486	7	1240	9.8%	27.53 [12.98, 58.38]
Creech et al	361	3007	10	997	16.0%	13.47 [7.15, 25.35]
Frenck et al	68	1131	11	1129	12.5%	6.50 [3.42, 12.36]
Walter et al.	228	1517	45	751	61.8%	2.78 [1.99, 3.87]
Total (95% CI)		8141		4117	100.0%	7.36 [5.76, 9.40]
Total events	993		73			

Heterogeneity: $Chi^2 = 48.52$, $df = 3$ ($P < 0.00001$); $I^2 = 94\%$
Test for overall effect: $Z = 15.99$ ($P < 0.00001$)

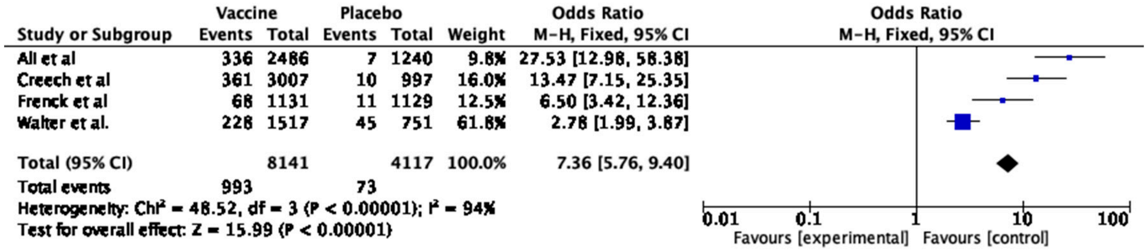

Study or Subgroup	Vaccine Events	Vaccine Total	Placebo Events	Placebo Total	Weight	Odds Ratio M-H, Fixed, 95% CI
Ali et al	484	2480	11	1222	15.6%	26.70 [14.62, 48.73]
Creech et al	511	3007	10	997	16.4%	20.21 [10.76, 37.95]
Frenck et al	56	1124	11	1117	13.8%	5.27 [2.75, 10.12]
Walter et al.	288	1514	38	747	54.2%	4.38 [3.09, 6.22]
Total (95% CI)		8125		4083	100.0%	10.58 [8.27, 13.55]
Total events	1339		70			

Heterogeneity: Chi² = 41.78, df = 3 (P < 0.00001); I² = 93%
Test for overall effect: Z = 18.72 (P < 0.00001)

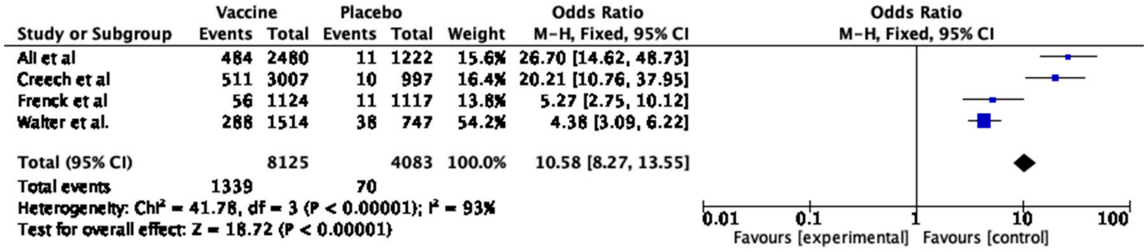

Figure S4 Swelling after dose 1 and 2

Study or Subgroup	Vaccine Events	Vaccine Total	Placebo Events	Placebo Total	Weight	Odds Ratio M-H, Fixed, 95% CI
Ali et al	403	2486	12	1240	20.8%	19.80 [11.10, 35.31]
Creech et al	361	3007	10	997	20.5%	13.47 [7.15, 25.35]
Frenck et al	79	1131	11	1129	15.9%	7.63 [4.04, 14.42]
Walter et al.	152	1517	23	751	42.9%	3.52 [2.25, 5.51]
Total (95% CI)		8141		4117	100.0%	9.59 [7.29, 12.62]
Total events	995		56			

Heterogeneity: Chi² = 26.85, df = 3 (P < 0.00001); I² = 89%
Test for overall effect: Z = 16.17 (P < 0.00001)

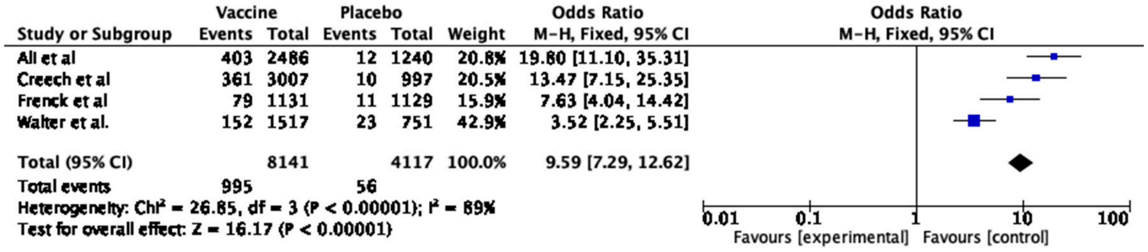

Study or Subgroup	Vaccine Events	Vaccine Total	Placebo Events	Placebo Total	Weight	Odds Ratio M-H, Fixed, 95% CI
Ali et al	508	2480	12	1222	21.1%	25.98 [14.59, 46.25]
Creech et al	571	3007	10	997	20.1%	23.14 [12.33, 43.42]
Frenck et al	56	1124	11	1117	17.3%	5.27 [2.75, 10.12]
Walter et al.	227	1514	22	747	41.4%	5.81 [3.72, 9.09]
Total (95% CI)		8125		4083	100.0%	13.47 [10.23, 17.73]
Total events	1362		55			

Heterogeneity: Chi² = 29.34, df = 3 (P < 0.00001); I² = 90%
Test for overall effect: Z = 18.54 (P < 0.00001)

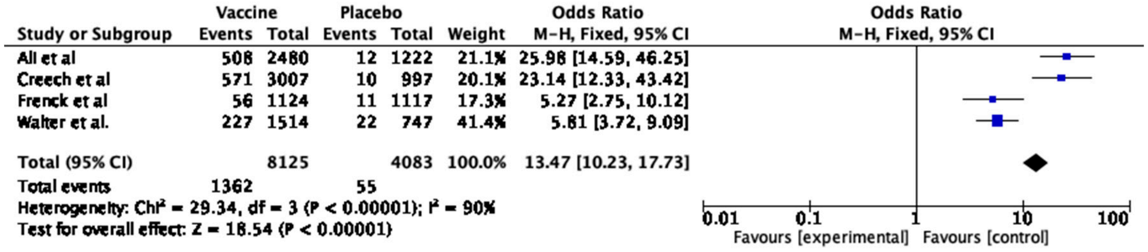

Figure S5 Fatigue after dose 1 and 2

Study or Subgroup	Vaccine Events	Vaccine Total	Placebo Events	Placebo Total	Weight	Odds Ratio M-H, Fixed, 95% CI
Ali et al	1191	2486	454	1240	31.7%	1.59 [1.38, 1.83]
Creech et al	1293	3007	339	997	29.1%	1.46 [1.26, 1.70]
Frenck et al	679	1131	463	1129	18.6%	2.16 [1.83, 2.56]
Walter et al.	516	1517	233	751	20.6%	1.15 [0.95, 1.38]
Total (95% CI)		8141		4117	100.0%	1.57 [1.45, 1.70]
Total events	3679		1489			

Heterogeneity: Chi² = 25.58, df = 3 (P < 0.0001); I² = 88%
Test for overall effect: Z = 11.15 (P < 0.00001)

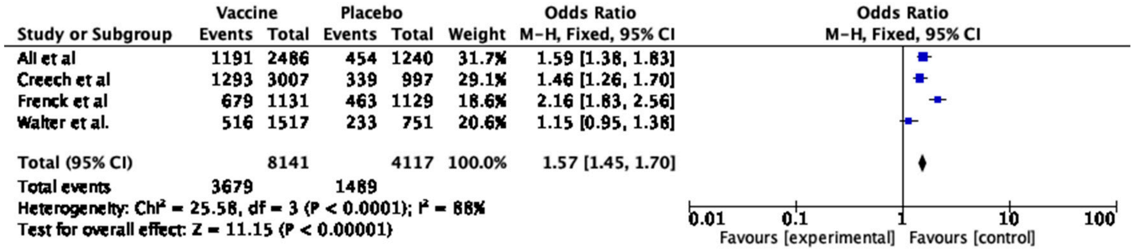

Study or Subgroup	Vaccine Events	Vaccine Total	Placebo Events	Placebo Total	Weight	Odds Ratio M-H, Fixed, 95% CI
Ali et al	1681	2480	353	1222	26.7%	5.18 [4.46, 6.02]
Creech et al	1995	3007	349	997	30.9%	3.66 [3.15, 4.25]
Frenck et al	742	1124	279	1117	16.7%	5.83 [4.86, 7.01]
Walter et al.	590	1514	179	747	25.7%	2.03 [1.66, 2.47]
Total (95% CI)		8125		4083	100.0%	4.01 [3.69, 4.36]
Total events	5008		1160			

Heterogeneity: Chi² = 74.75, df = 3 (P < 0.00001); I² = 96%
Test for overall effect: Z = 32.75 (P < 0.00001)

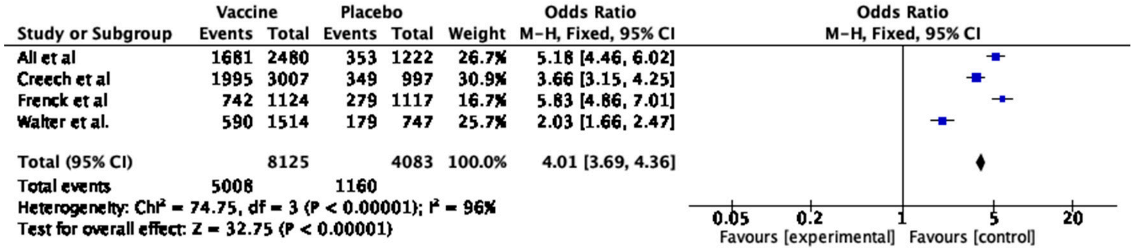

Figure S6 Headache after dose 1 and 2

Study or Subgroup	Vaccine Events	Vaccine Total	Placebo Events	Placebo Total	Weight	Odds Ratio M-H, Fixed, 95% CI
Ali et al	1109	2486	477	1240	33.9%	1.29 [1.12, 1.48]
Creech et al	932	3007	309	997	30.8%	1.00 [0.86, 1.17]
Frenck et al	622	1131	395	1129	17.1%	2.27 [1.92, 2.69]
Walter et al.	334	1517	180	751	18.1%	0.90 [0.73, 1.10]
Total (95% CI)		8141		4117	100.0%	1.30 [1.20, 1.41]
Total events	2997		1361			

Heterogeneity: $Chi^2 = 65.15$, df = 3 ($P < 0.00001$); $I^2 = 95\%$
Test for overall effect: Z = 6.28 ($P < 0.00001$)

Odds Ratio M-H, Fixed, 95% CI

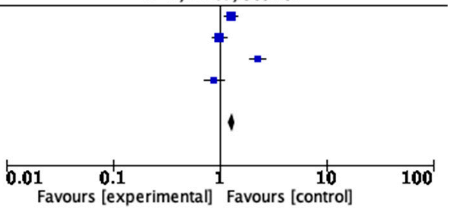

Study or Subgroup	Vaccine Events	Vaccine Total	Placebo Events	Placebo Total	Weight	Odds Ratio M-H, Fixed, 95% CI
Ali et al	1741	2480	370	1222	25.9%	5.42 [4.67, 6.30]
Creech et al	1624	3007	279	997	33.7%	3.02 [2.59, 3.53]
Frenck et al	731	1124	268	1117	16.5%	5.89 [4.90, 7.08]
Walter et al.	424	1514	142	747	24.0%	1.66 [1.34, 2.05]
Total (95% CI)		8125		4083	100.0%	3.79 [3.48, 4.13]
Total events	4520		1059			

Heterogeneity: $Chi^2 = 109.52$, df = 3 ($P < 0.00001$); $I^2 = 97\%$
Test for overall effect: Z = 30.62 ($P < 0.00001$)

Odds Ratio M-H, Fixed, 95% CI

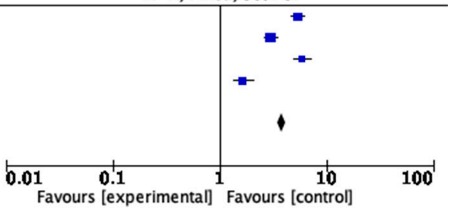

Figure S7 Nausea and vomiting after dose 1 and 2

Study or Subgroup	Vaccine Events	Vaccine Total	Placebo Events	Placebo Total	Weight	Odds Ratio M-H, Fixed, 95% CI
Ali et al	281	2486	110	1240	43.6%	1.31 [1.04, 1.65]
Creech et al	331	3007	110	997	49.3%	1.00 [0.79, 1.25]
Frenck et al	34	1131	11	1129	3.6%	3.15 [1.59, 6.25]
Walter et al.	30	1517	8	751	3.5%	1.87 [0.85, 4.11]
Total (95% CI)		8141		4117	100.0%	1.24 [1.06, 1.45]
Total events	676		239			

Heterogeneity: $Chi^2 = 11.88$, df = 3 ($P = 0.008$); $I^2 = 75\%$
Test for overall effect: Z = 2.74 ($P = 0.006$)

Odds Ratio M-H, Fixed, 95% CI

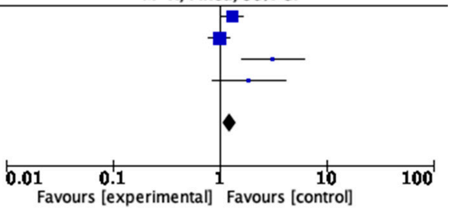

Study or Subgroup	Vaccine Events	Vaccine Total	Placebo Events	Placebo Total	Weight	Odds Ratio M-H, Fixed, 95% CI
Ali et al	593	2480	106	1222	44.6%	3.31 [2.66, 4.12]
Creech et al	722	3007	100	997	47.1%	2.83 [2.27, 3.54]
Frenck et al	34	1124	11	1117	4.4%	3.14 [1.58, 6.22]
Walter et al.	30	1514	7	747	3.8%	2.14 [0.93, 4.89]
Total (95% CI)		8125		4083	100.0%	3.03 [2.61, 3.52]
Total events	1379		224			

Heterogeneity: $Chi^2 = 1.65$, df = 3 ($P = 0.65$); $I^2 = 0\%$
Test for overall effect: Z = 14.49 ($P < 0.00001$)

Odds Ratio M-H, Fixed, 95% CI

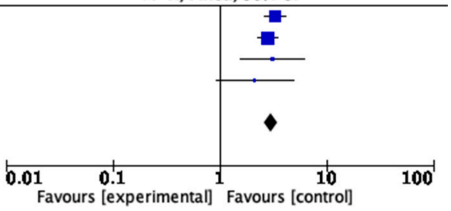

Table S2 Safety databases and surveillance systems

Name of resource	Year	Vaccine given	Vaccine type	Location	Age of participants	Number vaccinated
COVID-19 Vaccine Safety in Adolescents Aged 12-17 Years - United States, December 14, 2020-July 16, 2021	2021	Pfizer	RNA	USA	12 to 17	approx 8.9 million
COVID-19 Vaccine Safety in Adolescents Aged 12-17 Years - United States, December 14, 2020-July 16, 2021	2021	Pfizer	RNA	USA	12 to 17	approx 8.9 million
Coronavirus vaccine - weekly summary of Yellow Card reporting	2022	Pfizer, AstraZeneca, Moderna	RNA, Viral vector	United Kingdom	<18	First dose: 4.2 million<br>Second dose: 2.8 million
VigiAccess	2022	All COVID-19 vaccines, unable to determine vaccine type by database	Mixed	Worldwide	Age data brokend own into:<br>0-27 days, 28 days to 23 months, 2-11 years, 12-17 years, 18-44 years, 45-64 years, 65-74 years, >75 years	
Reported side effects following COVID-19 vaccination in Canada	2022	Pfizer	RNA	Canada	5 to 11, 12 to 17	12 to 17 year olds: Cumulative number of doses given to this age group:<br>5 to 11 year olds:<br>Number of doses given in this age group: 5,020,764
COVID-19 Vaccines, Overview of National Reporting Experience<br>Publication date: 17 February 2022 (Update #16)	2022	Pfizer	RNA	Ireland	5 to 11, 12 to 17	Total 875,000 doses in children between 5-17 years old
Adverse events following immunisation with COVID-19 vaccines: Safety Report #45 – 31 August 2022	2022	Pfizer	RNA	New Zealand	5 to 11, 12+	5-11: Dose 1: 267,314<br>Dose 2: 149,818<br>12 to 19 - data not availabe, instead data for 12+ presented by.
EudraVigilance	2022	Pfizer	RNA	EU / EEA	0-1 month, 2 months - 2 years, 3-11 years, 12-17 years	Not described in dashboard.
EudraVigilance	2022	Moderna	RNA	EU / EEA	0-1 month, 2 months - 2 years, 3-11 years, 12-17 years	Not described in dashboard.
EudraVigilance	2022	Astrazeneca	Viral vector	EU / EEA	0-1 month, 2 months - 2 years, 3-11 years, 12-17 years	Not described in dashboard.
EudraVigilance	2022	Janssen	Viral vector	EU / EEA	0-1 month, 2 months - 2 years, 3-11 years, 12-17 years	Not described in dashboard.
Moderna COVID-19 vaccine safety data - Adolescent participants	2022	Moderna	RNA	Australia	<20	Surveys completed:<br>Moderna dose 1: 5,352<br>Moderna dose 2: 4847
Pfizer COVID-19 vaccine safety data - Child participants	2022	Pfizer	RNA	Australia	5 to 11	Surveys complete:<br>Pfizer dose 1: 133,618<br>Pfizer dose 2: 82,340
Pfizer COVID-19 vaccine safety data - Adolescent participants	2022	Pfizer	RNA	Australia	<20	Surveys completed:<br>Pfizer dose 1: 231,093<br>Pfizer dose 2: 172,002<br>Pfizer dose 3 / booster : 35,155
VAERS	2022	Pfizer	RNA	USA		Not described in dashboard.
VAERS	2022	Moderna	RNA	USA		Not described in dashboard.
